# Clinical outcomes of left main coronary artery disease patients undergoing three different revascularization approaches

**DOI:** 10.1097/MD.0000000000009778

**Published:** 2018-02-16

**Authors:** Chieh-Shou Su, Yu-Wei Chen, Ching-Hui Shen, Tsun-Jui Liu, Yen Chang, Wen-Lieng Lee

**Affiliations:** a Cardiovascular Center, Taichung Veterans General Hospital, Taichung; b Institute of Clinical Medicine, and Department of Medicine, National Yang-Ming University School of Medicine, Taipei; c Division of Cardiology, Department of Internal Medicine, Taichung Veterans General Hospital Chiayi Branch, Chiayi; d Department of Anesthesiology, Taichung Veterans General Hospital, Taichung; e Department of Surgery; f Department of Medicine, National Yang Ming University School of Medicine, Taipei, Taiwan.

**Keywords:** conventional coronary artery bypass graft surgery, left main coronary artery disease, percutaneous coronary intervention, robot-assisted coronary artery bypass graft surgery

## Abstract

Significant unprotected left main (LM) coronary artery disease is frequently associated with severe multivessel disease and increased mortality and morbidity compared with non-LM coronary artery disease. This study compared the clinical outcomes of patients with LM disease who received percutaneous coronary intervention (PCI) with stenting, conventional coronary-artery bypass grafting (C-CABG), and robot-assisted CABG (R-CABG).

This retrospective study analyzed 472 consecutive LM disease patients who underwent three different revascularization approaches at a tertiary medical center between January 2005 and November 2013.

Of the 472 LM disease patients, 139 received R-CABG, 147 received C-CABG, and 186 received PCI. The need for target vessel revascularization (TVR) was highest in the PCI group. The R-CABG group had significantly lower rates of in-hospital and follow-up all-cause deaths compared with the other 2 groups (1.4% vs. 3.4% and 9.7%, *P* = .0058; 13.7% vs. 29.3% and 29.6%, *P* = .0023, respectively). Patients in the R-CABG group had significantly lower rates of intra-aortic balloon pump assistance, and shorter duration of ICU and total hospital stay compared to patients in the C-CABG group. However, revascularization modality, SYNTAX scores, and residual SYNTAX scores were not independent predictors of in-hospital or long-term mortality.

In this cohort of LM disease patients treated at a tertiary medical center, PCI is a reasonable choice in patients with less lesion complexity but who are older and have comorbidities. R-CABG is feasible in stable LM disease patients with high SYNTAX scores, and is an effective alternative to C-CABG in LM disease patients with few risk factors. However, revascularization modality per se was not a determinant for long-term mortality in our real-world practice.

## 1 Introduction

Significant left main (LM) coronary artery disease (CAD) occurs in 3% to 9% of patients undergoing coronary angiography.^[1,2]^ Current guidelines^[3–5]^ recommend coronary artery bypass grafting (CABG) as the criterion standard therapy for LM disease. Although percutaneous coronary intervention (PCI) has been shown to be an effective alternative for treating LM disease,^[6–9]^ there has been an increasing focus on minimally invasive endoscopic surgical techniques^[10–14]^ owing to advantages such as minimal operation wound, shorter hospital stay, faster recovery, and similar or even better clinical outcomes with these techniques compared with traditional surgeries. Robot-assisted CABG (R-CABG) has been proven to be safe and efficient for treating valvular and congenital heart diseases,^[15–18]^ as well as for revascularizing simple and complex CAD.^[19–22]^ However, to the best of our knowledge, there are currently no data comparing the treatment effects of R-CABG with conventional CABG (C-CABG) and PCI for LM disease. The purpose of this study was to compare clinical outcomes in LM patients treated with R-CABG, C-CABG, or PCI in real-world practice.

## 2 Methods and materials

This retrospective study recruited all consecutive patients with angiographically proven LM disease who underwent either R-CABG, C-CABG, or PCI at our institute between January 2005 and November 2013. Significant LM disease was defined as a >50% narrowing of the lumen diameter as determined by angiography. The choice of revascularization modality was mainly determined by guidelines, but also partially at the discretion of the attending physicians. As a rule, patients with proven LM disease and complex anatomy were recommended CABG as the first therapy or PCI as an alternative therapy if they declined CABG. Patients who chose surgery received either C-CABG or R-CABG depending on comorbidities, personal willingness, and financial status. The PCI, C-CABG, and R-CABG procedures were all carried out following the standard practices at this institute. Briefly, the 3 different revascularization approaches were performed as follows: R-CABG was performed using the Da Vinci robotic system and under general anesthesia. For this procedure, the cardiovascular surgeon harvested the left radial artery by endoscopy followed by wound closure, and then the left internal mammary artery (LIMA) inside the chest. Subsequently, pericardiotomy was performed to expose the native coronary arteries through 3 pencil-sized incisions along the left anterior axillary line over 2^nd^, 4^th^, and 6^th^ intercostal spaces. An incision about 2.5 to 3 cm long was then created over the 2^nd^ intercostal space near the sternal bone for the anastomosis of the harvested radial artery and LIMA grafts in end-to-side fashion. After this, a hand-sewn off-pump LIMA-LAD anastomosis in end-to-side mode and sequential LIMA-radial artery-grafts to diagonal artery, left circumflex, or posterior descending artery anastomoses, depending on lesion involvements, were performed via an 8-cm left anterolateral thoracotomy. The C-CABG was performed by traditional sternotomy under general anesthesia. Briefly, the cardiovascular surgeon harvested the LIMA and performed pericardiotomy to expose the coronary arteries via the median sternotomy. Then the surgeon harvested the left radial artery from the left forearm or superficial femoral vein from left femoral thigh as decided by the surgeon. After that, a hand-sewn LIMA-LAD anastomosis in end-to-side fashion and Y-anatomosis of LIMA-sequential radial artery grafts to diagonal artery, left circumflex obtuse marginal branches or posterior descending artery, depending on lesion involvements, were performed. The C-CABG procedure was performed on the beating heart or on the arrested heart. The percutaneous LM intervention was performed by experienced interventional cardiologists in our institute. The decision on 1-stent or 2-stent strategy for LM lesion was made by the operator according to the bifurcation classification, coronary flow of main and side branch, angulation, vessel dominance, and calcification during the index procedure. The decision of whether to use a drug-eluting stent (DES) or bare-metal stent (BMS) for LM PCI was also at the operator's discretion of the operators, and based on the lesion characteristics and the patient's financial status. No dedicated bifurcation stent was used, as it was not available in this institute. The final kissing technique of LM bifurcation post stenting was an essential step on all 2-stent cases except in those in whom balloon or wire passing failed. The treatment of all other non-LM lesions also followed the general principle to pursue complete revascularization. Medical records in the hospital database were retrospectively reviewed for the statistical analysis of baseline demographic data, in-hospital and long-term outcomes. The study protocol was reviewed and approved by the Institutional Review Board/Ethics Committee of Taichung Veterans General Hospital, Taichung, Taiwan.

## 3 Statistical analysis

Continuous variables are presented as mean ± standard deviations if normally distributed or median with interquartile range if not. Categorical variables are presented as numbers and percentages. The continuous variables were first tested for data distribution by the Kolmogorov-Smirnov test for each variable in each group. If the variables were normally distributed, they were analyzed by 1-way analysis of variance (ANOVA), and, if not normally distributed, by the Kruskal-Wallis test. Post-hoc analysis was analyzed by Dunn-Bonferroni test. Categorical variables were analyzed by *χ*^2^ test. A Kaplan-Meier survival curve was performed for long-term mortality. Logistic and Cox regression analyses were used to determine the independent factors associated with in-hospital and long-term follow-up mortalities. A *P* value <0.05 was considered statistically significant. All statistical analyses were performed using SPSS 19.0 (SPSS Inc., Chicago, IL) software.

## 4 Results

### 4.1 Baseline characteristics of all patients with LM diseases

**Figure 1:**
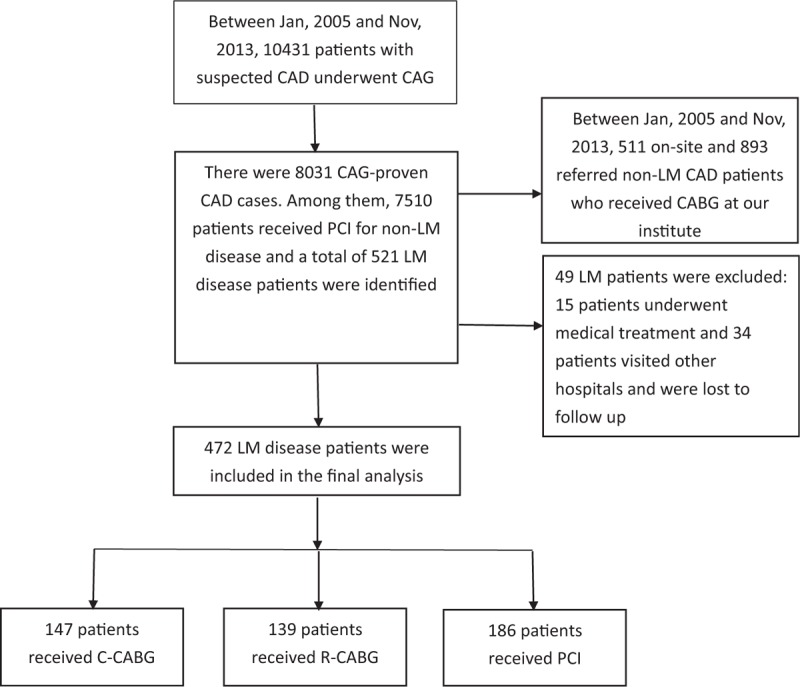
Flow chart of study design. A total of 472 patients with proven LM disease who received R-CABG, C-CABG, and PCI were included in the final analysis. C-CABG = conventional coronary artery bypass grafting, LM = left main, PCI = percutaneous coronary intervention, R-CABG = robot-assisted coronary artery bypass grafting.

Figure 1 is a flow chart of patients with proven LM disease who received R-CABG, C-CABG, and PCI, and who were included in the final analysis. Of the 521 patients with LM disease, 472 patients were included in the final analysis. The study population comprised 139 patients who received R-CABG, 147 patients who received C-CABG, and 186 patients who received PCI. The patients who received R-CABG all underwent complete robotic-assisted procedure without any conversion to median sternotomy. One hundred and fourteen of 147 (77.5%) patients in the C-CABG group underwent bypass surgery on the beating heart, and the radial artery was used for bypass graft in 130 of 147 (88.4%) patients. In the PCI group, 163 of 186 (86.7%) LM lesions were treated using the 2-stent strategy, and DES was used in all these patients. Of these 163 patients, the Culotte style for 2-stent strategy was utilized in 150 patients (92%), the DK crush style was used in 9 patients (5.5%), and the TAP technique was used in 4 patients (2.5%). Of the remaining 23 patients treated with the 1-stent strategy, 10 patients (43.5%) received DES, and the others received BMS. The baseline characteristics of all study patients are shown in Table 1. Patients in the R-CABG group were significantly younger, had less acute coronary syndrome (ACS), chronic renal disease (CKD), cardiogenic shock, lower serum creatinine and hemoglobin, and higher left ventricular ejection fraction (LVEF) as compared with the other 2 groups. Patients in the PCI group patients were significantly older, had lower SYNTAX and residual SYNTAX scores, and had a greater prevalence of previous PCI and CABG history compared to the other groups.

**Table 1:**
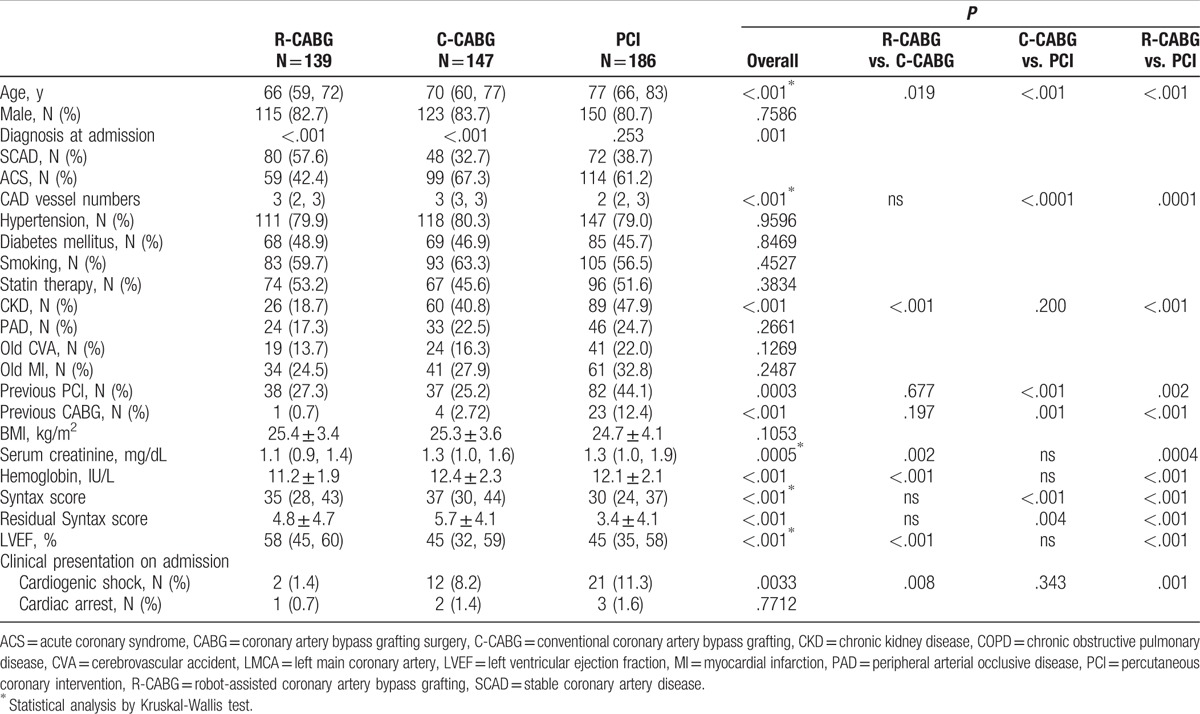
Demographic characteristics of LMCA disease patients in the 3 different revascularization groups.

### 4.2 In-hospital and long-term clinical outcomes

The observed in-hospital and long-term clinical outcomes are shown in Table 2. The need for intra-aortic balloon pump (IABP) assistance was highest in the C-CABG group and lowest in the PCI group. The duration of intensive care unit (ICU) stay, as well as the duration of in-hospital stay was longest in the C-CABG group. The incidence of in-hospital deaths was lowest in the R-CABG group, and there was no difference in the number of in-hospital deaths between the C-CABG and PCI groups.

**Table 2:**
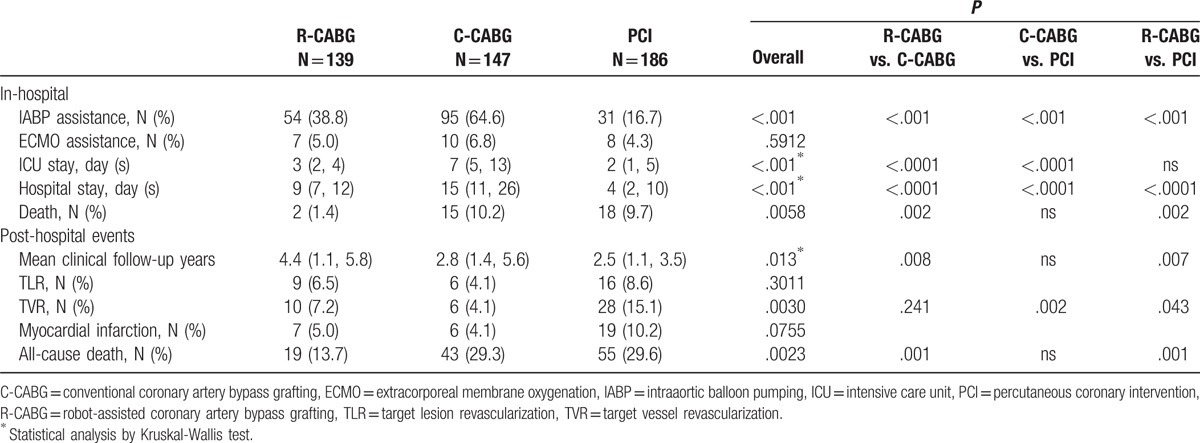
In-hospital and post-hospital clinical outcomes of patients with LMCA disease in the 3 different revascularization groups.

After hospital discharge, the 3 groups were followed up for a median duration of 4.4, 2.8, and 2.5 years, respectively (*P* = .013). There was no significant difference in the incidence of target lesion revascularization (TLR) and MI between the 3 groups. However, patients in the PCI group had a significantly higher incidence of TVR compared to the R-CABG and C-CABG groups. The total death rate in the R-CABG group was significantly lower compared to the C-CABG and PCI groups.

**Figure 2:**
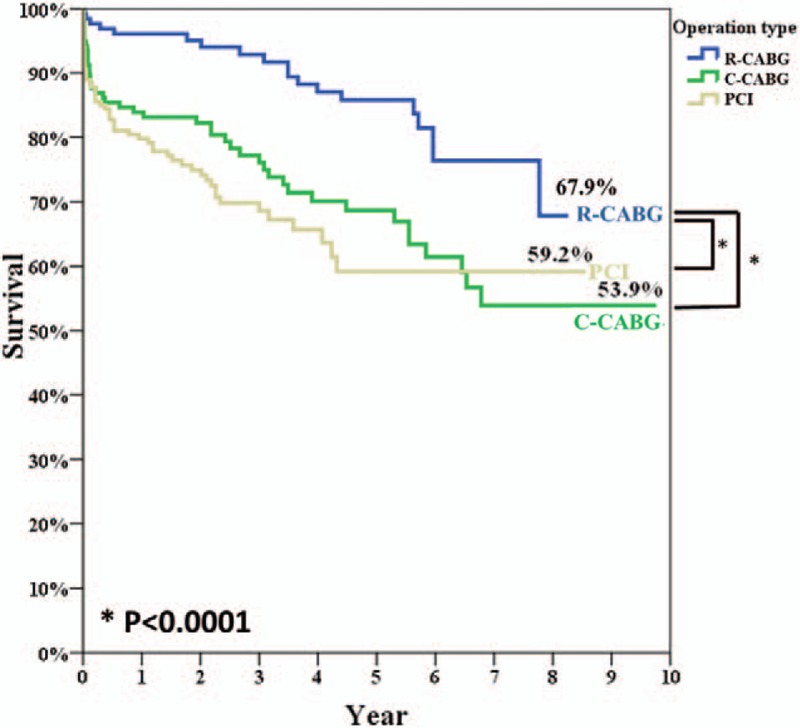
Survival rates after PCI (gray line) versus C-CABG (green line) and R-CABG (blue line) in post-hospital discharge clinical follow-up (59.2%, 53.9% vs. 67.9%, log-rank *P* < .0001). C-CABG = conventional coronary artery bypass grafting, PCI = percutaneous coronary intervention, R-CABG = robot-assisted coronary artery bypass grafting.

A Kaplan-Meier survival curve was used to analyze survival outcomes in the 3 groups (Fig. 2). The R-CABG group had significantly better outcomes than those of the other 2 groups, whereas there was no difference in outcomes between the C-CABG and PCI groups.

### 4.3 Clinical predictors for in-hospital and long-term mortality in all LM disease patients

Univariate and multivariate analyses were used to identify clinical predictors for in-hospital and long-term mortalities in LM disease patients who underwent different revascularizations (Tables 3 and 4). Multivariate logistic regression analysis showed that statin therapy and CKD were independent predictors of in-hospital mortality, whereas multivariate Cox regression analysis showed that age, diabetes mellitus (DM), LVEF, statin therapy, and CKD were independent predictors of long-term mortality. However, the revascularization modality per se was not an independent predictor for either in-hospital or long-term survival in our patient cohort. Neither SYNTAX score nor residual SYNTAX score was found to be independent risk factors for mortality in our cohort.

**Table 3:**
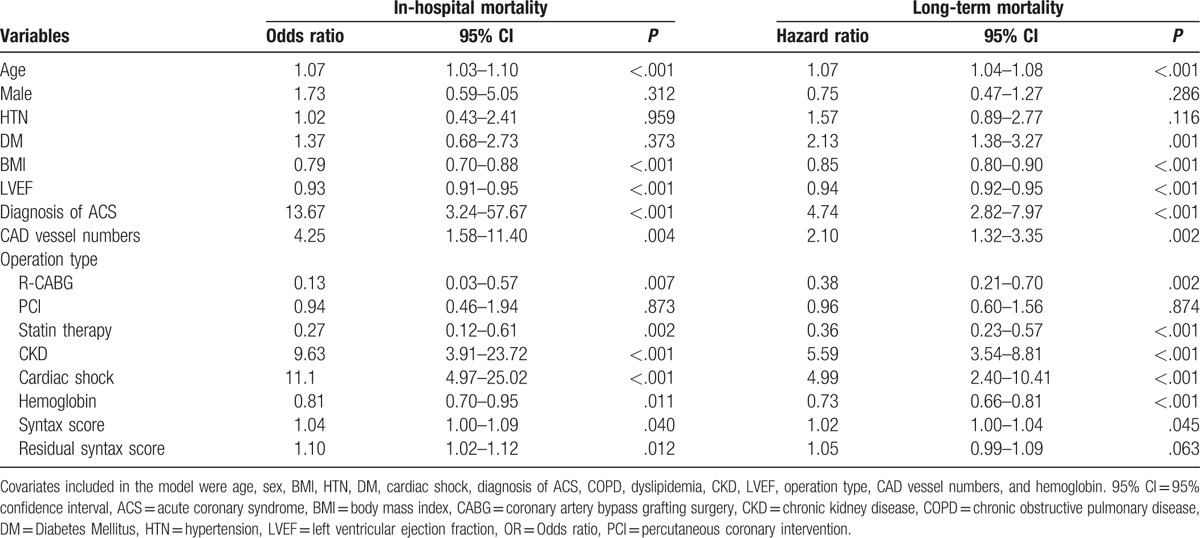
Univariate logistic and Cox regression analyses to identify predictors of in-hospital and long-term mortality of patients with LMCA disease.

**Table 4:**
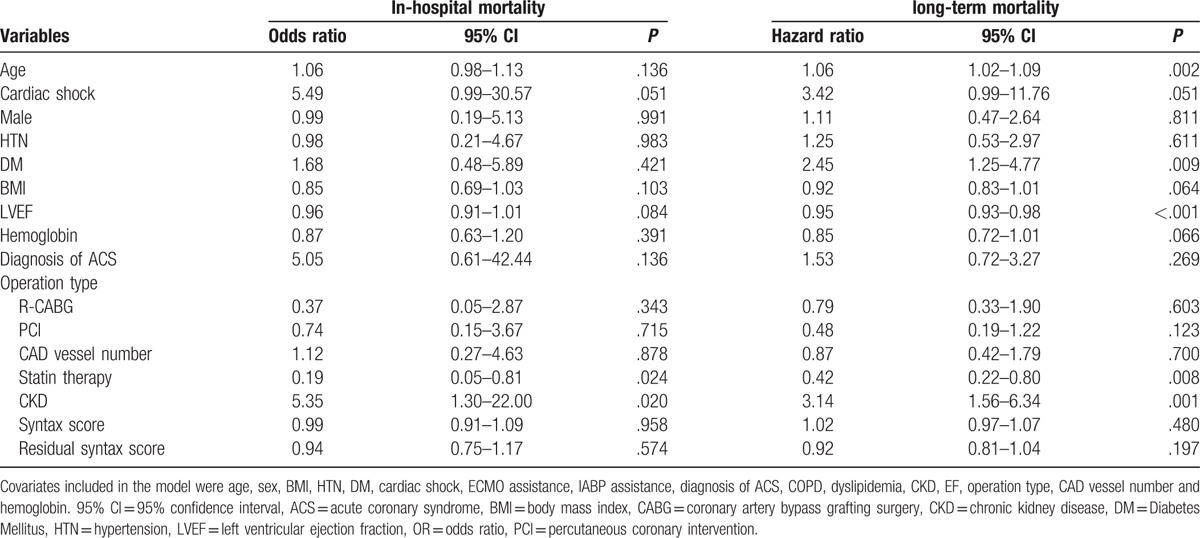
Multivariate logistic and Cox regression analyses to identify predictors of in-hospital mortality and long-term mortality of patients with LMCA disease.

## 5 Discussion

To the best of our knowledge, this is the first study to compare short- and long-term clinical outcomes of R-CABG, C-CABG, and PCI in the real-world treatment of LM disease. The main findings of the present study were that: PCI was associated with long-term survival equivalent to that of C-CABG, but had a higher TVR than R-CABG and C-CABG; R-CABG is feasible in high-SYNTAX score LM disease patients, which showed a better unadjusted long-term survival than C-CABG or PCI, but this could be attributed to fewer baseline comorbidities in this cohort; statin therapy and CKD were found to be independently associated with in-hospital mortality, whereas age, DM, LVEF, statin therapy, and CKD were independent predictors of long-term survival. However, the revascularization modality (operation type) per se was not an independent predictor for mortality in the present cohort.

Significant unprotected LM is regarded as the most prognostically important coronary lesion because it is frequently associated with severe multivessel disease and increased mortality and morbidity compared with non-LM coronary artery disease. Age, LVEF, DM, dyslipidemia, CKD, and clinical presentation of ACS have been shown to be poor predictors of mortality in patients with LM disease.^[23,24]^ Recent studies^[6–9]^ have shown that PCI might be a valid alternative for treating LM disease in the DES era, especially for patients with simple LM lesions, or in the acute setting.^[25,26]^ However, the risks of TVR and TLR were consistently higher in patients treated with PCI compared to the CABG group. Recently, the SYNTAX score has been suggested to be a useful parameter in predicting clinical outcomes of LM disease patients undergoing revascularization.^[27,28]^ The incidence of major adverse cardiac and cerebrovascular events (MACCE) was higher in the PCI group when the SYNTAX score was >33, and this was mostly driven by a higher need for revascularization,^[7,9,29]^ although the death rate did not differ. This finding was substantiated by the latest EXCEL study,^[30]^ whose findings were consistent with the results of the C-CABG and PCI groups in our present real-world study. In our current retrospective study, PCI was found to be associated with higher TVR compared to the C-CABG group, but in-hospital and long-term survival rates in the 2 groups were similar. The similarity in survival could be because of the lower SYNTAX score and less diseased CAD vessel numbers in the PCI group. However, PCI group patients were significantly older, had higher prevalence of previous PCI, CABG history, and higher serum creatinine, which might have offset the advantage of lower SYNTAX score at study entry. Our PCI practice was consistent with the guidelines, favoring patients with less lesion complexity, but who were older and had more comorbidities. Our findings suggested that PCI remains a good choice for selected patients with LM diseases.

With innovations in surgical devices and techniques, open-wound surgeries have gradually been replaced by endoscopic techniques in recent decades, as these are associated with shorter ICU and total hospital stays, lower blood transfusion requirements, less postoperative complications, and better post-operative quality of life. Robot-assisted surgery has been proven to be effective, efficacious, and has increasingly been used during the past few decades.^[10–14]^ In the field of cardiovascular treatment, the desire for a minimally invasive method of revascularizing the heart has also led to the widespread use of PCI even though in complex cases of CAD, CABG has been shown to be more efficacious, with lower rates of mortality, MI, and reintervention in mid- and long-term follow-up. During the past 2 decades, there has been a significant increase worldwide in the use of robot-assisted cardiovascular surgery using the Da Vinci system, which combines the advantages of 2 revascularization methods to provide smaller wounds, less rib retraction, reduction in pain, and faster return to normal activities with a positive impact on the quality of life,^[20,31]^ in patients with congenital^[17,18]^ and valvular heart diseases, ^[15,16]^ and CAD.^[19–22]^ However, R-CABG was mostly used for treating simple but not complex CAD because it is more time-consuming and technically demanding than C-CABG. Currie et al^[20]^ reported comparable long-term graft patency rates and quality of life in patients treated with R-CABG compared with those treated with C-CABG. However, their patients were younger, and had lower co-morbidities, and mostly had single vessel disease. Cavallaro et al^[22]^ evaluated a large cohort of patients from a national database and found that R-CABG was most often performed in cases with a single CABG graft, more stable background condition, a lower numbers of co-morbidities, and lower postoperative complication rates compared to C-CABG. Moreover, R-CABG was considered to be an effective alternative for treating isolated left anterior descending artery lesions alone or as a hybrid revascularization in combination with PCI with good short-term clinical and angiographic results.^[19]^ Our current study also showed that R-CABG was used mostly in patients with stable CAD or fewer comorbidities (higher LVEF, less cardiogenic shock, and better renal function). However, 42.6% of R-CABG was done in patients presenting with ACS, a median of 3 diseased coronary arteries, and a median SYNTAX score of 35 at our institute. The good outcomes seen in these patients suggested that in contrast with earlier reports,^[20,21]^ R-CABG was feasible in patients with complex and advanced lesions. In our patient cohort, R-CABG generated a better unadjusted in-hospital and long-term survival than either C-CABG or PCI, shorter hospital stay than C-CABG, and less TVR than PCI. However, the better outcomes might be attributed to more stable patient status and fewer background comorbidities at entry. Multivariate analysis ruled out revascularization modality per se as an independent predictor for long-term mortality. Based on our data showing that R-CABG could significantly shorten the duration of ICU, and total hospital stay and minimize postoperative patient discomfort and care, we suggested that R-CABG is an effective and efficient alternative for surgically revascularizing high SYNTAX score patients beyond the C-CABG.

Residual SYNTAX Score (RSS) and the term of incomplete revascularization after coronary revascularization were found to be associated with adverse outcomes,^[32–34]^ with RSS ≥ 8 predicting all-cause mortality, revascularization, and stent thrombosis. The RSS in our R-CABG patients was comparable with that in the SYNTAX study, arguing the good results could be reproduced by the much less invasive R-CABG in real-world practice. However, the RSS in our PCI patients was significantly lower than those of C-CABG or R-CABG patients. However, this could be because of lower SS at the baseline. Furthermore, our multivariable analysis ruled out either baseline or residual SS as a predictor of mortality.

In conclusion, in this cohort of LM disease patients treated at a tertiary medical center, patients who underwent PCI had more revascularization during follow-up, but similar in-hospital and follow-up mortalities compared to those underwent C-CABG. R-CABG is feasible in stable patients with high-SYNTAX score LM disease and could be an effective and efficient alternative for revascularizing these patients with less background risk factors.

### 5.1 Study limitations

This study had some important limitations. First, this was an observational, retrospective, and nonrandomized study, and therefore subject to all the limitations inherent in the study design. Second, the choice or assignment of revascularization modality in the present study was based on the attending physician's recommendation, the patient's and family's choice and their financial capability, rather than random assignment. Thus, there were significant differences in comorbidities between the groups at the study entry. However, our study tried to correct for these by performing multivariate Cox regression analysis. As R-CABG demands special patient requirements and PCI is suitable for less complex lesions, we think our study population and design reflected real-world practice, and thus our conclusions are relevant to the general clinical settings. However, the study population in each group of our study was relatively small in size. Our study conclusions should be confirmed with larger randomized trials to better define the long-term outcomes of R-CABG compared with C-CABG and PCI in patients with LM disease.
